# Improved prognosis for recurrent epithelial ovarian cancer by early diagnosis and 125I seeds implantation during suboptimal secondary cytoreductive surgery: a case report and literature review

**DOI:** 10.1186/s13048-020-00744-2

**Published:** 2020-11-30

**Authors:** Lin Xiao, Junying Tang, Wenbo Li, Xuexun Xu, Hao Zhang

**Affiliations:** 1 Department of Gynecology, the First Affiliated Hospital of Chongqing Medical University, Chongqing, 400016 China; 2 Department of Nuclear medicine, the First Affiliated Hospital of Chongqing Medical University, Chongqing, 400016 China

**Keywords:** Recurrent epithelial ovarian cancer, Secondary cytoreductive surgery, 125I seeds implantation

## Abstract

**Background:**

Epithelial ovarian cancer (EOC) has the worst prognosis in all of gynecologic malignant tumors because of its high recurrence and eventually chemo-resistance. Early diagnosis of recurrence is crucial to avoid diffuse dissemination. Failure of traditional treatment in recurrent epithelial ovarian cancer remains a challenge for clinicians. On the other hand, ^125^I brachytherapy has been accepted as a useful and hopeful treatment for multiple advanced cancers in recent years. However, its success in advanced epithelial ovarian cancer is limited. Here we report a case of recurrent ovarian cancer who had been early diagnosis of isolated recurrence and successfully treated with ^125^I seeds implantation during suboptimal cytoreductive surgery.

**Case presentation:**

A 59-year-old woman presented with recurrent epithelial ovarian cancer who have had a history of ovarian cancer stage IIIB and an R0 resection had been achieved nearly 2 years before presented in our hospital. She underwent suboptimal secondary cytoreductive surgery after four cycles of chemotherapy with little effectiveness and severe chemotherapy-related side effects. Approximately 70% of the cancer-bulk was resected during surgery. For residual lesion which fixed around the right ureter and right external iliac vessel, ^125^I seeds implantation was performed. Postoperatively, the patient was treated with two cycles of combination chemotherapy with paclitaxel and carboplatin. The patient was free of disease at 26 months’ follow-up period.

**Conclusion:**

In recurrent EOC patients with unresectable isolated lesion, salvage 125I seeds implantation are feasible and may contribute to survival.

## Background

In all of the gynecologic malignant tumors, epithelial ovarian cancer (EOC) has the worst prognosis. The 5-year survival rate of 36% in stage III and 17% in stage IV [1] is depressing. The low survival rate of advanced EOC is mainly due to its high recurrence rate and eventually the emergence of diffuse dissemination and chemo-resistance.

The treatments for recurrent epithelial ovarian cancer include secondary cytoreductive surgery, chemotherapy, targeted therapy, immunotherapy, radiotherapy, and so on. Unfortunately, the effectiveness remains poor. As a result, additional and alternative therapeutic strategies to improve outcomes are urgently needed.

In the last decade, 125I brachytherapy has been accepted as a useful and minimally invasive treatment for many advanced cancers with significant efficacy and as a salvage therapy. However, its successful application in ovarian cancer is little and limited at present.

Here we present a case of recurrent epithelial ovarian cancer successfully early diagnosed and treated with 125I seeds implantation during suboptimal cytoreductive surgery. We felt the report of the patient would be of highly interest.

## Case presentation

**Fig. 1:**
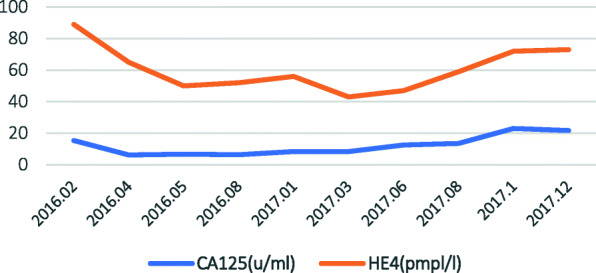
Timeline showing CA125 and HE4 levels after primary cytoreductive surgery

A 59-year-old female patient with a medical history of ovarian cancer presented in our hospital (the First Affiliated Hospital of Chongqing Medical University, Gynecological Oncology Unit) in December 2017. The patient had been submitted to cytoreductive surgery nearly 2 years before (January 2016). At that time, she complained of lower abdominal pain and bilateral pelvic mass with elevated serum CA125 level of 778.1 u/ml and HE4 level of 609 pmol/L. A primary cytoreductive surgery with removal of total hysterectomy with bilateral adnexectomy, omentectomy, pelvic and para-aortic lymph node dissection and other visible disease was performed. At that moment, an R0 resection was achieved. According to surgery and histopathological examination, the surgical-pathologic staging was medium to poorly differentiated epithelial serous ovarian adenocarcinoma IIIB (FIGO, 2014). Postoperatively, the patient was submitted to six cycles of adjuvant platinum- and taxane-based chemotherapy. The follow-up revealed no recurrent manifestation for nearly the next 2 years. The lowest value of CA-125 was 6.2u/ml during the period of chemotherapy and follow-up period (Fig. 1).

**Fig. 2:**
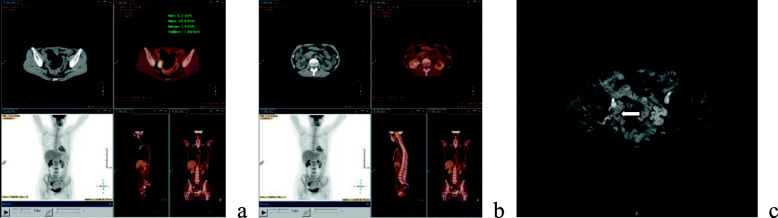
Recurrent lesion: **a** PET-CT lesion recurred from ovarian cancer at right pelvic cavity. **b** Mild hydronephrosis. **c** MRI lesion recurred from ovarian cancer after four cycles of chemotherapy

Nearly 2 years later after primary surgery (December 2017), the patient developed slight elevated serum CA125 with the value of 21.7 u/ml and HE4 73 pmol/L, both of which still in the normal range. However, the Positron Emission Tomography-Computed Tomography (PET-CT) (Fig. 2) showed a locoregional recurrence of about 3-4 cm in diameter mass located at right pelvic cavity with mild hydronephrosis.

Firstly, the patient chose chemotherapy due to fear of surgery complication. Considering of platinum sensitive, she was submitted to four cycles of adjuvant platinum- and taxane-based chemotherapy. However, the effectiveness was not good according to the magnetic resonance imaging (MRI) (Fig. 2) and CA125 level. At the same time, the chemotherapy-related side effects were severe.

After a discussion of multidisciplinary treatment and informed consent of the patient, secondary surgery was performed in April 2018, by a combined approach with gynecology, gastrointestinal surgery, and nuclear medicine.

Intraoperatively, the presence of the isolated recurrence around the right ureter and right external iliac vessel was confirmed. Part of the tumor was densely adherent and invaded to small intestine. To avoid major bleeding, partial tumor resection with 10 cm part of ileum resection and side-to-side ileoileostomy was performed. Approximately 70% of the cancer-bulk was resected during surgery. For residual lesion which fixed around the right ureter and right external iliac vessel, 125I seeds implantation was performed. 18 G implantation needles were inserted directly into the target lesions avoiding puncture of large blood vessels and nearby ureter, a turntable gun was then used to place 125I seeds into recurrent tumors. Then seeds were released 0.5-1 cm apart upon withdrawing the needles.

The postoperative course was uneventful and the patient was discharged in the seventh postoperative day.

Histopathological results confirmed the recurrence with malignant cells that invaded the small intestine with negative resection margins.

Postoperatively, the patient was submitted to two cycles of adjuvant platinum-based chemotherapy. At more than 2 years of follow-up, the patient is free of any local or distant recurrent disease and asymptomatic.

**Fig. 3:**
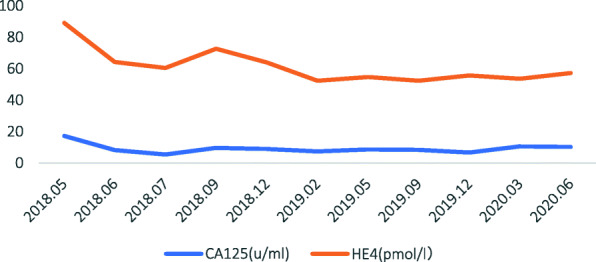
Timeline showing CA125 and HE4 levels after second cytoreductive surgery and seeding implantation

The range of CA125 level during follow-up was below 15u/ml (Fig. 3).

**Fig. 4:**
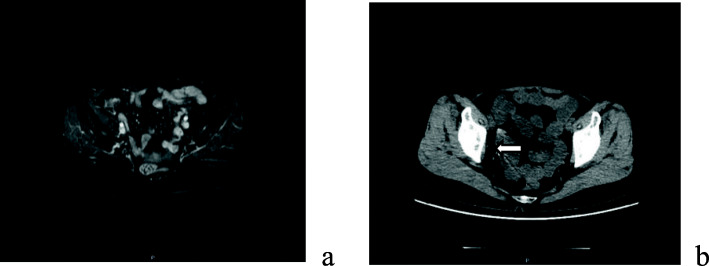
Postoperative image in June 2019: a. MRI showed no obvious local lesion. b. CT showed tumor disappeared, with only radioactive seeds remaining

Postoperative MRI and computed tomography (CT) were performed as Fig. 4.

## Discussion

Epithelial ovarian cancer (EOC) always remains the most lethal gynecologic malignancy, guideline-recommended treatments for advanced ovarian cancer is primary debulking surgery followed by platinum-based chemotherapy. However, relapse would almost unavoidable. Conventional treatment for recurrent EOC is chemotherapy and/or cytoreduction. Other researches in ovarian cancer in progress include multiline or dose-dense chemotherapy and targeted agents such as PARP inhibitors, Anti-angiogenic agents, immunotherapies.

Dose-dense weekly paclitaxel scheme in epithelial ovarian cancer has obtained remarkable attention in the past decade. However, the efficacy of chemotherapy alone is limited. Chen, Wei-Chun et al. [2] analyze the response to dose-dense chemotherapy of weekly paclitaxel and 3-weekly carboplatin in 16 patients with recurrent ovarian cancer, the median PFS of all patients were 10.9 months (range 4.3–40.5). The median disease-free survival (DFS) was 5.6 months (range 1.2–34.2).

Target therapy mainly including PARP inhibitors and anti-angiogenic agents which demonstrating efficacy with improved PFS and OS are mainly used as maintenance therapy for patients with recurrent ovarian cancer [3–6]. Meanwhile, Tumor burden is a potential marker of PARP inhibitor effects in ovarian cancer [7].

Immune therapy which targeting the programmed cell death protein 1 (PD-1)/programmed death-ligand 1 (PD-L1) mechanism of tumor immune evasion is a promising field of ovarian cancer therapy. In recent years, these inhibitors are being investigated in clinical trials. Inconsistent results have been obtained between studies. Some reports demonstrate a survival benefit with increased PD-L1 tumor expression, while others have shown a negative result [8, 9].

For EOC, radiotherapy is not a routinely therapy. In recent years, studies have reported some favorable outcomes in patients with recurrent epithelial ovarian cancer treated with radiotherapy. Chang, JS et al. [10] included patients with recurrent epithelial ovarian cancer eligible for involved-field radiation therapy (IFRT) either during diagnosis of the recurrence or after salvage therapies. The overall and complete response rates were 85.7 and 50%, respectively. After a median follow-up of 28 (range, 17–42) months, the median PFS was 7 months. The 2-year PFS rate was 39.3%. In the study of a multicenter, retrospective study (MITO RT-01), Macchia G et al. [11] included 261 patients with metastatic, persistent, recurrent ovarian cancer (MPR-OC), carrying a total of 449 lesions treated by stereotactic body radiotherapy (SBRT). complete response (CR), partial response (PR) and stable disease (SD) to SBRT were observed in 291 (65.2%), 106 (23.8%), and 33 (7.4%) lesions.

The role of secondary cytoreductive surgery (SCS) in recurrent epithelial ovarian cancer is yet controversial. For platinum-sensitive recurrent ovarian cancer, SCS increases survival rate. In a case-control study, Marchetti, C et al. [12] included 46 platinum-sensitive recurrent EOC patients. Twenty-three women undergone SCS and followed by platinum-based chemotherapy and olaparib maintenance. The other 23 women only received medical treatment. Median time to first subsequent therapy (TFST) was significantly longer in the SCS + medical group than in the medical group. Also, SCS + medical patients had better post-recurrence survival (PRS), with a 3-year PRS of 79% in SCS + medical group versus 42% in medical group. However, Coleman RL et al. [13] randomly assigned patients with recurrent ovarian cancer with platinum-sensitive in the Gynecologic Oncology Group (GOG)-213 trial, a phase 3 randomized prospective trial. The median OS was 50.6 months in patients with surgery and 64.7 months in patients without surgery (HR = 1.29; 95% CI = 0.97–1.72; *p* = 0.08) while median PFS was 18.9 months in patients with surgery and 16.2 months in patients without surgery (HR = 0.82; 95% CI = 0.66–1.01). They concluded that secondary cytoreductive surgery followed by chemotherapy did not result in longer overall survival than chemotherapy alone.

The postoperative residual tumor mass is the most important prognostic factor. Indication to Secondary cytoreductive surgery should be individualized. Early diagnosis of recurrence is the key of the possibility of surgery and complete cytoreduction would improve the prognosis.

One advantage of our case is early diagnosis of recurrence. Regular follow-up and early diagnosis of recurrence is of great importance for EOC after primary therapy. If the recurrence is isolated, there maybe the chance of secondary cytoreductive surgery and relatively good prognosis. Unfortunately, cases with isolated recurrences are not common. Many cases have disseminated lesions at the time of diagnosis. The rigorous surveillance of patients after primary treatment is a challeng in clinical practice. We think the suspicion of recurrence should be considered once the serum CA125 levels elevated to more than 15 U/ml or two times of its lowest level. In this case report, PET-CT discovered metastatic foci in early-stage even if the serum tumor marker remains in normal range. Highly alertness of recurrence in the follow-up of EOC patients is important. With the help of high-quality image, clinicians could correctly monitor patients, distinguish relapse patterns and preform correct management management [14].

Most of the recurrent lesions were near or adhered by even infiltrated surrounded important organs such as ureter, vagina, cyst, intestine or rectum. Sometimes tumors could not be removed because their removal would cause severe functional disability or life-threatening bleeding. Since reported by Brunschwig [15] in 1948, the pelvic exenteration (PE) has become an important method to treat pelvic malignancies. However, such management has remained controversial because of its severe functional disability or heavy hemorrhage especially when the tumor fixed to the pelvic sidewall. New treatment strategies for unremovable lesion in secondary cytoreductive surgery for recurrent ovarian cancer are needed.

Another particularity and highlight of our case are partially tumor resection with salvage 125I seeds implantation which did not interfere with the function of the patient and received good effects.

Compared to the other kinds of radiotherapy, 125I brachytherapy has several advantages. Its benefit is boosted by natural increases in local dose. 125I seed local treatment can reduce the tumor burden, relieve local symptoms and improve quality of life of patients. Early in 1991, Iodine-125 interstitial implants as salvage therapy for recurrent gynecologic malignancies including one ovarian carcinoma has been reported [16]. Now 125I brachytherapy has increasingly been used for other sites of disease, such as central nervous system, head and neck tumors, lung, hepatic and pancreatic cancer and so on. Efficacy and safety of iodine-125 radioactive seeds brachytherapy has been approved [17, 18].

In 1999, there has been American Brachytherapy Society (ABS) recommendations for the clinical quality assurance and guidelines of permanent prostate brachytherapy with 125I [19]. In 2018, Chinses expert consensus statement on computed tomography-guided 125I radioactive seeds permanent interstitial brachytherapy has been developed [20].

In radiotherapy-naive patients with unresectable isolated recurrent gynecologic malignancies, 125I implants are feasible and may possibly contribute to survival [21]. Unlikely as cervical carcinoma or endometrial carcinoma, radiotherapy is not usually been used primarily in patients with ovarian epithelium cancer. As a result, 125I brachytherapy is a hopeful therapy for recurrent ovarian cancer.

The success of 125I brachytherapy is dependent on and the size of tumors and the accurate placement of radioactive seeds [22]. Usually, all the 125I seeds implantation was performed with CT or ultrasound guidance. In our case, 125I seed implanted directly under the vision of operation. On the one hand, tumor burden is reduced by surgery; on the other hand, 125I implantation is more accurate and safer. Combination of surgery and 125I seeds implantation improved the prognosis for recurrent epithelial ovarian cancer.

## Conclusion

We hope that this case report, demonstrating prolonged survival by early diagnosis of recurrence and 125I seeds implantation during suboptimal secondary cytoreductive surgery with little cost, will serve to strengthen alertness of recurrence in the follow-up of EOC patients and interest in the 125I brachytherapy of unremovable recurrent EOC.

## Data Availability

The data used or analyzed are all included in this published article.
